# Factors associated with patient dissatisfaction after unicompartmental knee arthroplasty: a retrospective cohort analysis of 978 patients

**DOI:** 10.1186/s12891-026-09904-9

**Published:** 2026-05-08

**Authors:** Cong Wang, Guorong Zhang, Yihui Liu, Desheng Chen, Zhigang Bai, Yuqi Liang

**Affiliations:** 1 Department of Joint Surgery, People’s Hospital of Ningxia Hui Autonomous Region, Ningxia Medical University, Yinchuan, 750002 China; 2 Medical Education Management Office, People’s Hospital of Ningxia Hui Autonomous Region, Ningxia Medical University, Yinchuan, 750002 China; 3 Clinical Medical College, Qinghai University, Xining, 810016 China; 4 Department of Anesthesiology and Operating Room, People’s Hospital of Ningxia Hui Autonomous Region, Ningxia Medical University, Yinchuan, 750002 China

**Keywords:** Unicompartmental Knee Arthroplasty, Patient Satisfaction, Pain, Logistic Regression Analysis

## Abstract

**Background:**

Medial compartment knee osteoarthritis is common. Unicompartmental knee arthroplasty (UKA) offers a bone-preserving alternative to total knee replacement. Despite its advantages, patient dissatisfaction remains a concern. This study aimed to identify factors associated with dissatisfaction following UKA.

**Methods:**

Methods A retrospective analysis was conducted on 978 patients who underwent UKA between January 1, 2017 and December 31, 2023. Postoperative outcome data were collected via telephone and outpatient follow-up (mean follow up 49.6 months, range 20–102 months). Only unilateral UKA cases were included. Dissatisfaction was defined as a response of “neutral”, “dissatisfied”, or “very dissatisfied” on a 5 point Likert scale.

**Results:**

Among 978 patients, 811 (82.9%) were satisfied and 167 (17.1%) were dissatisfied. Multivariate logistic regression identified kneeling difficulty (OR=0.128, 95%CI: 0.079–0.206), stair-related pain (OR=8.783, 95%CI: 5.588–13.803), walking pain (OR=9.463, 95%CI: 5.591–16.014), and knee instability (OR=3.988, 95%CI: 2.179–7.298) as independent factors associated with dissatisfaction.

**Conclusions:**

Patient dissatisfaction after UKA is independently associated with walking pain, stair-related pain, knee instability, and inability to kneel fully. Surgeon experience, prosthesis type, and preoperative patellar morphology (Wiberg III) were associated with satisfaction in univariate analysis but were not identified as independent factors in multivariate regression.Preoperative counseling should address these modifiable factors.

**Trial registration:**

Retrospectively registered. No trial registration number is applicable.

## Introduction

Knee osteoarthritis (KOA) is a prevalent degenerative disease among middle-aged and elderly individuals, severely impacting patients’ quality of life and imposing a substantial socio-economic burden.Research indicates that KOA does not invariably involve all three compartments of the knee; approximately 85% of cases primarily affect the medial compartment [1], Consequently, unicompartmental knee arthroplasty (UKA) presents a viable alternative for a subset of patients in managing knee arthritis [2], Favored by many orthopedic surgeons for its advantages such as minimal invasiveness, bone preservation, rapid recovery, and potentially superior knee kinematics [3, 4], studies suggest that around 47% of patients with KOA are candidates for UKA. Nonetheless, the utilization rate of UKA remains low, at only 5–8% [5, 6].Despite continuous advancements in surgical techniques and instrumentation, a discrepancy in patient satisfaction persists between UKA and total knee arthroplasty (TKA). Studies report that the lifetime revision risk for UKA is approximately twice that of TKA (UKA ranging from 3.7% to 40.4% vs. TKA from 1.6% to 22.4%) [7], This higher revision rate may be one factor influencing the choice of procedure. Importantly, revision and dissatisfaction are distinct concepts; a patient may be satisfied despite a revision, or dissatisfied without implant failure.The reasons for UKA failure are multifaceted, including progression of arthritis in the contralateral compartment, aseptic loosening, infection, and mobile bearing dislocation. These complications not only affect surgical success rates but are also significant contributors to postoperative patient dissatisfaction.Patient dissatisfaction following UKA has been reported in 10–30% of patients across various series [8], indicating that a notable proportion of patients do not achieve the expected outcome.Traditionally, satisfaction better reflects patients’ subjective judgment, avoiding false-negative results associated with purely functional knee scores. This is because some patients may also report unexplained discomfort, such as that caused by weather changes. Therefore, subjective satisfaction serves as an excellent objective indicator.**P**revious studies have identified multiple factors associated with satisfaction after UKA, including preoperative functional status, postoperative pain relief, range of motion, patient expectations, and absence of residual symptoms. However, most of these studies have focused on Western populations, with limited attention to culturally specific functional demands such as kneeling or squatting. Furthermore, few large-sample studies have simultaneously examined the impact of multiple postoperative symptoms on satisfaction in a single cohort.Despite extensive research on UKA designs (fixed vs. mobile bearing, cemented vs. cementless, manual vs. robotic-assisted), no single variant has demonstrated universal superiority in patient satisfaction [9]. Robotic-assisted UKA may improve satisfaction compared to manual techniques (OR = 1.72), but the heterogeneity of satisfaction measurement tools remains a methodological challenge [10]. Most existing satisfaction studies originate from Western cohorts, leaving a gap in region-specific analyses for non-Western populations.Currently, research specifically investigating patient satisfaction following UKA, particularly within distinct regional populations, is lacking. This study systematically analyzes clinical data from patients who underwent UKA at the Department of Joint Surgery, Ningxia Hui Autonomous Region People’s Hospital, between January 1, 2017, and December 31, 2023. It aims to identify the key factors influencing postoperative satisfaction in this cohort, thereby providing targeted guidance for preoperative counseling, patient selection, surgical technique optimization, and postoperative rehabilitation.We hypothesized that (1) postoperative pain (walking and stair-related) and functional limitations (kneeling difficulty, instability) would be independently associated with patient dissatisfaction, and (2) preoperative factors such as surgeon experience, prosthesis type, and patellar morphology would also influence satisfaction rates.The primary outcome was patient satisfaction (dichotomized as satisfied vs. dissatisfied). Secondary outcomes included postoperative symptoms (walking pain, stair-related pain, knee instability, kneeling ability) and functional scores (HSS, WOMAC, FJS-12).

## Materials and methods

### Study population

This retrospective study analyzed the clinical data of patients who underwent UKA at the Department of Joint Surgery, Ningxia Hui Autonomous Region People’s Hospital, between January 1, 2017, and December 31, 2023. The study protocol was reviewed and approved by the Hospital Ethics Committee (Approval No. 2025-WJW-004).

### Inclusion and exclusion criteria

#### Inclusion criteria

Diagnosis of single-compartment knee osteoarthritis, predominantly affecting the medial compartment, confirmed by clinical and imaging examinations; first-time UKA treatment; availability of complete preoperative knee function scores and imaging data; absence of severe cardiovascular, cerebrovascular, hepatic, or renal diseases.

#### Exclusion criteria 

Exclusion Criteria: Concurrent severe cardiovascular or cerebrovascular diseases (e.g., New York Heart Association class III-IV heart failure), or hepatic/renal insufficiency (e.g., end-stage renal disease); inflammatory joint diseases such as rheumatoid arthritis; psychiatric disorders or cognitive impairment precluding follow-up cooperation; patients with bilateral UKA, our center followed up without identifying any cases of concurrent bilateral UKA [11].

**Fig. 1:**
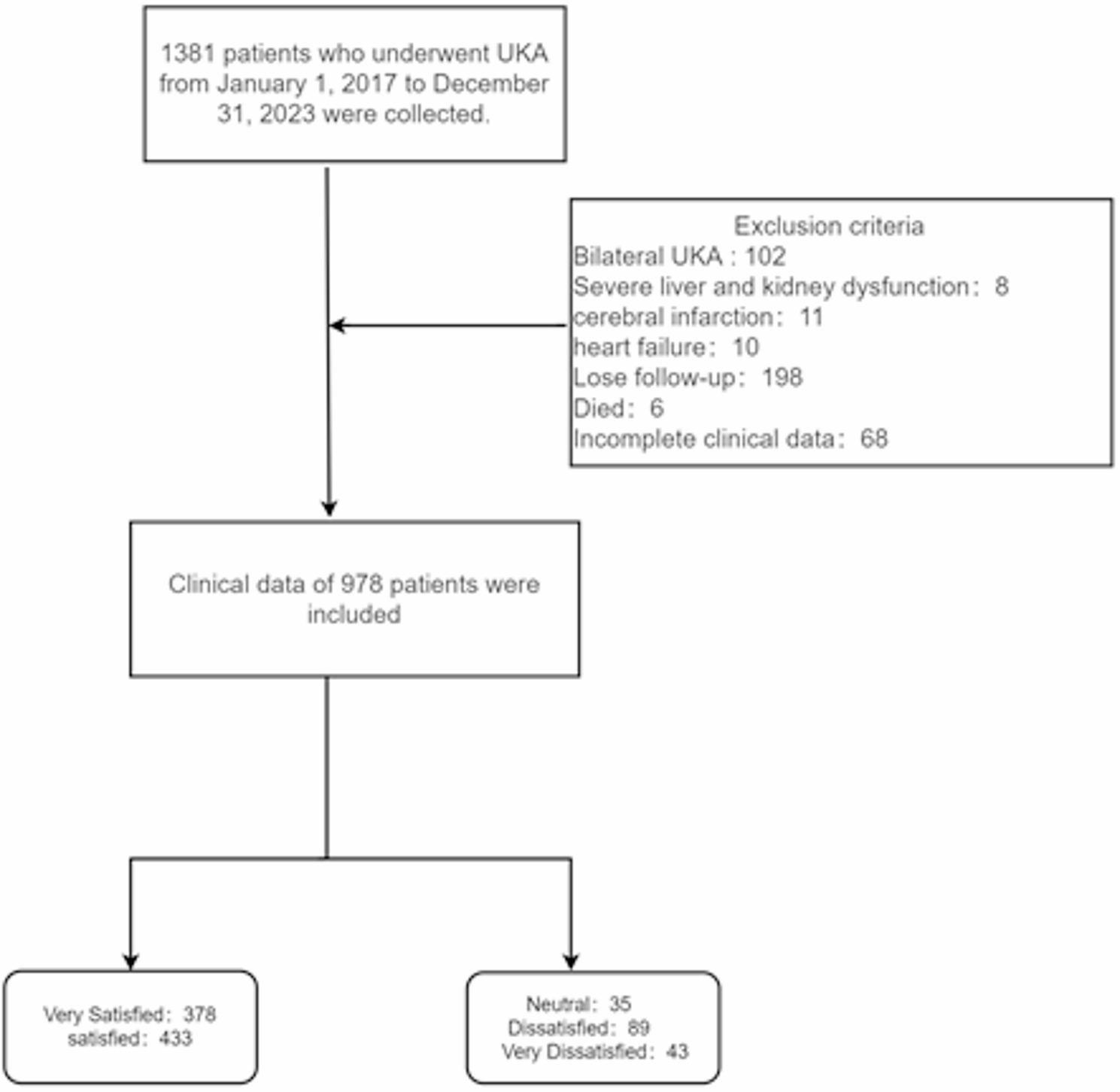
UKA satisfied patient screening flow chart

Based on these criteria, 978 patients were included: 276 males and 702 females. Age ranged from 44 to 88 years (mean: 64.41 ± 7.92 years). Follow-up duration ranged from 20 to 102 months (mean: 49.56 ± 19.36 months). Regarding prosthesis type: 592 received a mobile-bearing prosthesis, and 386 received a fixed-bearing prosthesis.(Fig. 1).

## Research methods

### Surgical technique

Four senior arthroplasty surgeons (> 10 years experience, > 50 UKA cases each) performed all procedures using a standardized medial parapatellar approach. Tibial preparation used an intramedullary or extramedullary guide (surgeon‘s preference) to achieve a horizontal cut perpendicular to the mechanical axis with 5–7° posterior slope. Femoral preparation used an intramedullary guide. Trial components were placed to assess gap balance and ligament tension; medial collateral ligament was preserved. Adjustments were made for asymmetric gaps or tightness. Final cemented fixation was used (Link^®^ Sled for fixed-bearing; Zimmer Biomet Oxford for mobile-bearing), achieving ≥ 0° extension and ≥ 120° flexion.

### Assessment parameters

Data were collected through outpatient reviews and telephone follow-ups, which specifically included: demographic information (age, gender, body mass index, and education level), symptom duration, preoperative patellar Wiberg classification, prosthesis type, complications, Hip-knee-ankle angle (HKA) 3–5 days after operation. and Posterior Tibial Slope (PTS), as well as functional scores at the final follow-up—the Hospital for Special Surgery Knee Score (HSS), the Western Ontario and McMaster Universities Osteoarthritis Index (WOMAC), and the Forgotten Joint Score-12 (FJS-12). The assessment of knee instability was primarily based on patients’ reports of giving way, buckling, or a sense of instability during daily activities, as recorded in follow-up interviews. Standardized physical stress tests for instability were not routinely performed.Kneeling ability was assessed by asking patients: “Are you able to kneel fully on the operated knee without discomfort?” (yes/no).

#### Wiberg classification

There are two physicians evaluated the patella using 90° axial X-ray films, knee CT scans and MRI images according to the Wiberg classification system. Wiberg Type I: Both medial and lateral patellar articular surfaces are essentially symmetrical (I); Wiberg Type II: The lateral articular surface is larger than the medial one (Group II); Wiberg Type III: The medial articular surface is extremely small or absent (Group III).

#### Satisfaction assessment 

Satisfaction was assessed using a 5-point Likert scale with the following question: “Overall, how satisfied are you with the outcome of your knee replacement surgery?” Response options were: 1 = very satisfied, 2 = satisfied, 3 = neutral, 4 = dissatisfied, 5 = very dissatisfied. Responses of “very satisfied” and “satisfied” were defined as “satisfied”; responses of “neutral,” “dissatisfied,” and “very dissatisfied” were defined as “dissatisfied” for the primary analysis.Neutral responses were combined with dissatisfied because they represent a lack of positive endorsement of the surgical outcome.As satisfaction was patient-reported, blinding was not relevant.

### Statistical analysis

Data analysis was performed using SPSS 26.0. Continuous variables are presented as mean ± standard deviation; after normality confirmation by the Shapiro-Wilk test, intergroup comparisons used the t-test with Cohen’s d and 95% CI. Categorical variables are presented as number (%) and were compared using the χ² test with φ or Cramer’s V. Inter-observer reliability for radiographic measurements was assessed using intraclass correlation coefficients (ICC). Multicollinearity was assessed using variance inflation factors (VIF); all VIF values were below 2, indicating no significant collinearity. Variables with *P* < 0.05 in univariate analysis were entered into a binary logistic regression model (Enter method). Odds ratios (OR) and their 95% CI are reported. With 167 dissatisfied patients and 5 variables in the final model, the sample size satisfied the recommended minimum of 10 events per variable. A P-value < 0.05 was considered statistically significant. No a priori sample size calculation was performed (consecutive retrospective cohort). Only patients with complete data were included (complete case analysis); no imputation was performed.

## Results

### Patient satisfaction distribution and overview

A total of 978 patients were included(Loss to follow-up rate: 14.3%).No significant differences in baseline characteristics were found between patients with complete vs. incomplete follow-up (data not shown) .Among them, 811 (82.9%, 95% CI: 80.4%–85.2%) were satisfied with the treatment outcome, and 167 (17.1%) were dissatisfied. The ICC values for PTS was 0.954 ,*P* < 0.001(95% CI: 0.919–0.974) ,HKA was 0.890 ,*P* < 0.001 (95% CI: 0.812–0.936), respectively, indicating good to excellent agreement.Effect size analysis indicated large effects for the HSS score (d = 3.88) and WOMAC score (d = 2.45. Stair-related pain (φ = 0.44), walking pain (φ = 0.34), and the ability to kneel fully (φ = 0.36) showed medium effect sizes, constituting the main clinical symptoms and functional limitations contributing to dissatisfaction. Age also showed a medium effect size (φ = 0.27), indicating significantly higher satisfaction among elderly patients (> 60 years).(Table 1).

**Table 1:** Comparison of continuous data between two groups of patients

	Satisfied(811)	Dissatisfied(167)	T	*P*	95%CI	Cohen's d
Follow-up time(months)	49.32±19.63	50.76±17.94	-0.877	0.117	-4.671,1.786	-0.07
Symptom duration(months)	6.12±2.84	5.99±3.02	0.542	0.588	-0.346,0.611	0.05
WOMAC	44.53±12.31	76.94±17.09	-28.803	<0.001	-34.629,-30.206	2.45
HSS	89.99±4.73	67.60±9.29	45.708	<0.001	21.424,23.346	3.88
FJS-12	78.22±4.72	76.45±5.46	4.304	<0.001	0.966,2.586	0.35
HKA	174.22±2.22	173.54±1.85	4.167	<0.001	0.318,1.039	0.33
PTS	8.61±2.27	8.62±2.27	-0.014	0.989	-0.381,0.376	0

### Comparison of general characteristics between groups

Univariate analysis revealed significant differences (*P* < 0.05) between the groups in the following variables: Patellar Wiberg classification (χ² = 13.596, *p* = 0.001, Cramer’s V = 0.12): Type III was associated with the highest dissatisfaction rate. Prosthesis type (χ² = 6.721, *p* = 0.01, Phi φ = 0.08): Satisfaction was significantly higher in the mobile-bearing group. Surgeon (χ² =9.284, *p* = 0.01, Cramer’s V = 0.21): Patient satisfaction varied among different surgeons, with one surgeon’s group showing the highest satisfaction proportion. Age (χ² =70.732, *p* < 0.001, Phi φ = 0.27): Satisfaction was significantly higher in patients > 60 years old.(Table 2).

**Table 2:** Comparison of categorical variables between two groups of patients

		Satisfied(811)	Dissatisfied(167)	χ²	*p*	Cramer's V/Phi φ
gender	Men	228(28.11%)	48(28.74%)	0.27	0.869	0.01
	Women	583(71.89)	119(71.26%)			
wiberg	1	280(34.53%)	42(25.15%)	13.596	0.001	0.12
	2	420(51.79%)	85(50.90%)			
	3	111(13.68%)	40(23.95%)			
side	right	481(59.31%)	86(51.48%)	3.469	0.063	0.06
	left	330(40.69%)	81(48.52%)			
Prosthesis type	fixed	476(58.70%)	116(69.46%)	6.721	0.01	0.08
	movable	335(41.3%)	51(30.54%)			
Education level	junior school	708(87.30%)	146(87.42%)	0.002	0.969	0.001
	High school and above	103(12.70%)	21(12.58%)			
surgeon	a	358(44.14%)	20(11.98%)	42.021	<0.001	0.21
	b	117(14.43%)	62(37.12%)			
	c	138(17.02%)	45(26.95%)			
	d	198(24.41%)	40(23.95%)			
Age(year)	>60	573(70.65%)	61(36.53%)	70.732	<0.001	0.27
	≤60	238(29.35%)	106(63.47%)			
BMI	normal	262(32.31%)	63(37.72%)	3.725	0.155	0.06
	overweight	421(51.91%)	73(43.72%)			
	Obese	128(15.78%)	31(18.56%)			
kneeling	yes	546(67.32%)	35(20.96%)	123.456	<0.001	0.36
	no	265(32.68%)	132(79.04%)			
Stair-related pain	yes	102(12.58%)	99(59.28%)	185.001	<0.001	0.44
	no	709(87.42%)	68(40.72%)			
Walking pain	yes	84(10.36%)	72(43.11%)	110.832	<0.001	0.34
	no	727(89.64%)	95(56.89%)			
Knee Instability	yes	58(7.15%)	42(25.15%)	48.869	<0.001	0.22
	no	753(92.85%)	125(74.85%)			
ASA	2	600(73.98%)	116(69.46%)	1.444	0.230	0.04
	3,4	211(26.02%)	51(30.54%)			

### Knee function outcome indicators

Significant differences were found between the satisfied and dissatisfied groups in: Ability to kneel fully (χ² =123.456, *p* < 0.001, Phi φ = 0.36); Stair-related pain (χ² =185.001, *p* < 0.001, Phi φ = 0.44); Walking pain (χ² =110.832, *p* < 0.001, Phi φ = 0.34); Knee instability (χ² =48.869, *p* < 0.001, Phi φ = 0.22). Furthermore, the dissatisfied group had a significantly higher WOMAC score (t =-13.233, *p* < 0.001, 95% CI: -14.142, -8.799, d = 2.45) and significantly lower HSS score (t = 46.859, *p* < 0.001, 95% CI: 4.855, 6.682, d = 3.88) and FJS-12 score (t = 4.304, *p* < 0.001, 95% CI: 0.966, 2.586, d = 0.35). The dissatisfied group also had a significantly smaller HKA angle (t = 4.167, *p* < 0.001, 95% CI: 0.318, 1.039, d = 0.33).(Table 1).

### Other indicators

There was no statistically significant association between satisfaction and the following variables: gender (χ² = 0.027, df = 1, *p* = 0.869), surgical side (χ² = 3.469, df = 1, *p* = 0.063), education level (χ² = 0.002, df = 1, *p* = 0.969), posterior tibial slope (t = -0.014, df = 976, *p* = 0.989), symptom duration (t = 0.542, df = 976, *p* = 0.588), follow-up time (t = -0.877, df = 976, *p* = 0.117), BMI (χ² = 3.725, df = 2, *p* = 0.155), or ASA classification (χ² = 1.444, df = 1, *p* = 0.230).(Tables 1 and 2).

### Distribution of patient-reported postoperative symptoms

**Fig. 2:**
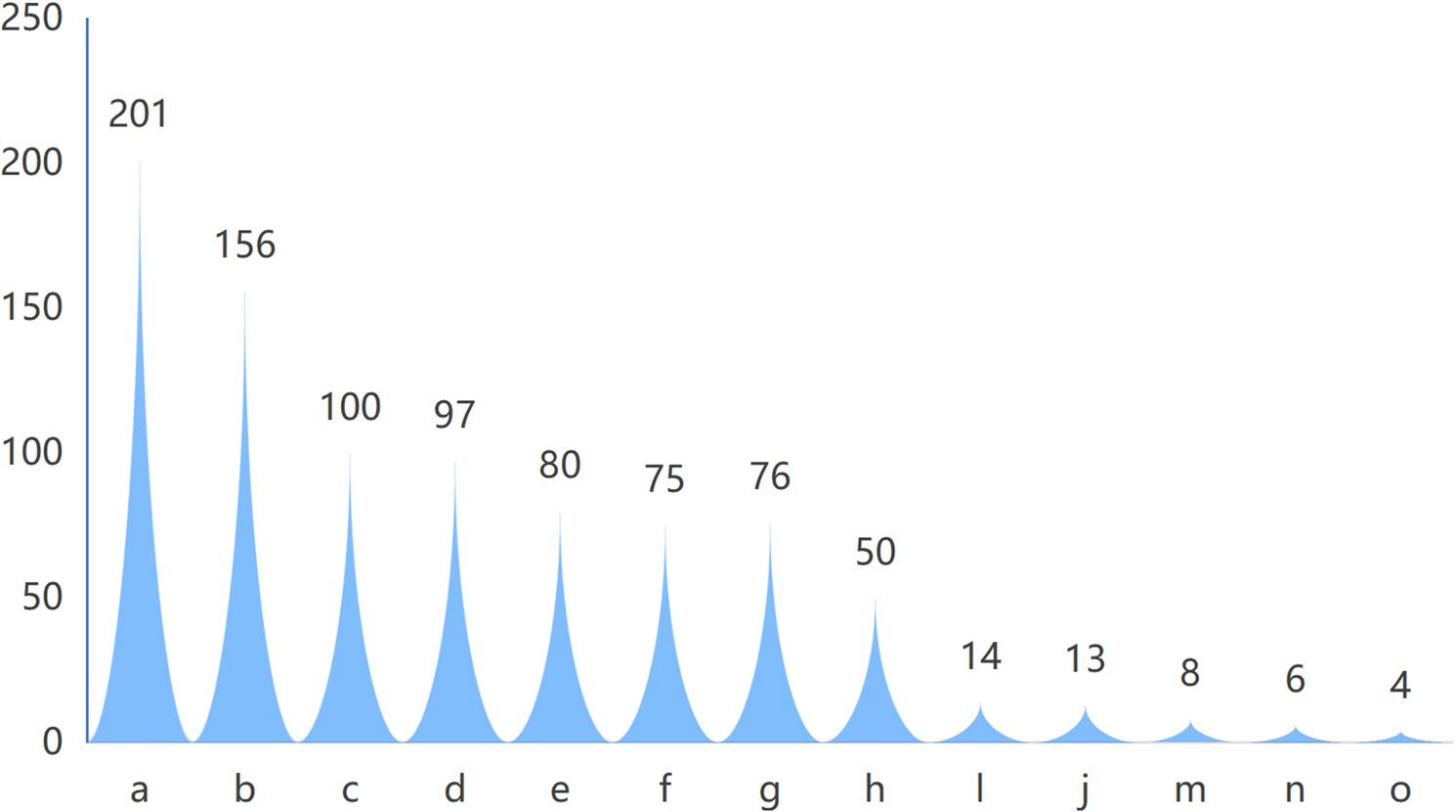
Postoperative patient complication distribution map. **a**: Pain During Stair Climbing/Descending(20.55%) **b**:Walking Pain(15.95%) **c**:Gait Instability(10.22%) d：Lateral Compartment Pain(9.9%) e：Other Factors(8.2%) **f**：Weather-related Discomfort(7.7%) **g**：Knee Stiffness((7.7%)) **h**：Swelling(5.1%) ** I**：Polyethylene Liner Dislocation Distribution(1.4%) ** j**：Revision(1.3%) **m**：Loosening(0.8%) **n**:Infection(0.6%) **o**：Periprosthetic Fracture(0.4%)Some patients reported multiple symptoms

The most commonly reported postoperative symptoms, in descending order of frequency, were: stair-related pain (*n* = 201, 20.55%), walking pain (*n* = 156, 15.95%), knee instability (*n* = 100, 10.22%), and lateral compartment pain (*n* = 68, 7.7%), .(Fig. 2).

### Multivariate logistic regression analysis

Variables with *P* < 0.05 in the univariate analysis were entered into the multivariate logistic regression model. The results identified the following as independent risk factors for dissatisfaction after UKA: Kneeling ability (OR = 0.128, 95%CI: 0.079–0.206),Walking pain (OR = 9.463,95%CI:5.591–16.014), Knee instability(OR = 3.988, 95%CI:2.179–7.298), and Stair-related pain(OR = 8.783, 95%CI:5.588–13.803). Notably, an OR < 1 for kneeling ability indicates that being able to kneel fully is a strong protective factor for satisfaction (OR = 0.128).Surgeon, prosthesis type, and Wiberg classification were significant in univariate analysis but became non-significant when postoperative symptoms were entered into the multivariate model, suggesting their effects on satisfaction are mediated through these symptoms.(Table 3).

**Table 3:** Logistic regression analysis results

	OR	SE	95% CI	*P*
Age	0.783	0.233	0.496,1.236	0.294
kneeling	0.128	0.244	0.079,0.206	<0.001
knee instability	3.988	0.308	2.179,7.298	<0.001
Stair-related pain	8.783	0.231	5.588,13.803	<0.001
walking pain	9.463	0.268	5.591,16.014	<0.001

## Discussion

Based on the analysis of clinical data from 978 UKA patients, this study found a postoperative satisfaction rate of 82.9%.Our observed satisfaction rate of 82.9% falls within the range reported in the literature. Annapareddy et al. reported a “very satisfied” rate of 75.2% in an Indian UKA cohort at a minimum 3-year follow-up [12]. The lower rate in that study may reflect their more stringent outcome definition, whereas we included both “very satisfied” and “satisfied” responses. Using data from the National Joint Registry for England and Wales, The satisfaction rate at 2 years postoperatively among 1,638 patients was 82.6%, which is consistent with our reported findings [13]. Dissatisfaction following UKA was closely associated with factors including the surgeon, prosthesis type, patellar morphology, age, kneeling ability, stair-related pain, walking pain, and knee instability. Most importantly, multivariate analysis identified kneeling difficulty, stair-related pain, walking pain, and knee instability as independent risk factors for dissatisfaction.Our univariate analysis revealed significant variation in satisfaction rates among surgeons, with Surgeon A’s cohort demonstrating the highest proportion of satisfied patients (Table 2). While this study was not designed to compare specific technical nuances, possible contributing factors may include differences in surgical approach, soft-tissue balancing technique, implant positioning preferences, or volume of UKA performed. Further prospective studies with standardized technique documentation are needed to elucidate which technical aspects most strongly influence patient-reported outcomes.

### Influence of surgical technique and prosthesis on satisfaction

Research has identified inherent varus deformity in 17% of female and 32% of male patients in Asian populations, with studies suggesting that mild varus alignment post-total knee arthroplasty (TKA) may correlate with better functional outcomes [14], For medial UKA, a postoperative varus alignment of 1–4° is associated with optimal functional results and implant survival [15].This target is not universal. The acceptable degree of varus correction is highly dependent on the patient’s preoperative constitutional alignment [16].This is supported by the work of Slaven et al., who reported that well-functioning UKA patients demonstrated approximately 4° of mild varus mechanical alignment at a 10-year follow-up [17], In our study, the dissatisfied group exhibited a smaller hip-knee-ankle (HKA) angle, a difference that was statistically significant but with a low effect size, and this variable was not an independent factor in the multivariate analysis. This suggests a complex and potentially non-linear relationship between lower limb alignment and patient satisfaction, warranting further investigation.While 85% of osteoarthritis cases primarily involve the medial compartment [1], the disease process often represents a broader degenerative progression. Consequently, after addressing the medial compartment, abnormal pressure changes in the lateral compartment become a critical factor affecting overall knee contact stress and serve as a biomechanical basis for postoperative pain and instability [18], Therefore, within the boundaries of established indications for UKA [19], achieving a patient-specific, moderate alignment correction may be more conducive to enhancing satisfaction.Our results (Table 2) revealed significant variation in patient satisfaction among different surgeon groups, a finding consistent with arthroplasty registry data highlighting the importance of surgical experience and procedural volume on UKA outcomes [20, 21], We acknowledge that the observed surgeon effect may be confounded by unmeasured factors such as case volume, years of experience, and patient selection, and no causal inference can be drawn.This highlights that both case volume accumulation and progression along the learning curve are equally important for improving surgical outcomes. Prosthesis stability is crucial for activities requiring high degrees of knee flexion, such as deep squatting. Previous studies have confirmed that with meticulous surgical technique, Asian patients can achieve good functional results following medial UKA [22]. Our study observed that satisfaction and perceived range of motion were lower with fixed-bearing prostheses compared to mobile-bearing designs. This may be attributed to the mobile-bearing design philosophy, which more closely replicates natural knee kinematics and has been associated with favorable long-term wear characteristics in biomechanical studies [23]. However, the direct link between polyethylene wear and early patient satisfaction remains uncertain.Furthermore, studies indicate that fixed-bearing UKA may increase lateral compartment pressure, whereas mobile-bearing designs better approximate physiological knee loading [24], However, given the retrospective nature of our study, the choice of prosthesis may have been subject to selection bias based on patient indications. This observation requires validation through prospective studies.

### Influence of patient factors on satisfaction

The impact of age on postoperative satisfaction following UKA is multifaceted. The univariate analysis in this study indicated that younger patients (age ≤ 60 years) reported significantly lower satisfaction than their older counterparts (age > 60 years), a finding consistent with some existing literature. For instance, Kleeblad et al. [25]observed that among UKA patients satisfied with their ability to return to sports, the satisfaction rate was higher in the 70-year-old age group (93.1%) compared to the 55-year-old group (77.8%). A meta-analysis also suggested that early return to physical activity postoperatively may be associated with better outcomes [26]. However, in the multivariate analysis of our study, age was not identified as an independent risk factor for dissatisfaction (*P* = 0.294). This suggests that the lower satisfaction observed in younger patients may not be directly attributable to age per se, but rather mediated indirectly through other age-correlated factors. Younger patients typically have higher activity levels and greater functional expectations [27], which may render them more sensitive to residual postoperative pain or functional limitations (such as stair-related or walking pain), thereby reducing their overall satisfaction. In other words, age likely serves as a surrogate marker for patient expectations and activity demands, whereas satisfaction is more directly influenced by the achievement of the specific functional states corresponding to those expectations.Concerning body mass index (BMI), traditional perspectives, such as the Scott criteria, often regard severe obesity (e.g., BMI > 35–40) as a relative contraindication for UKA, primarily due to concerns about implant survival under high mechanical loads. Our findings align with recent studies [28, 29].Showing no significant association between BMI and patient-reported satisfaction in the early postoperative period. This indicates that UKA remains an effective treatment option for obese patients in terms of pain relief and functional improvement. Nevertheless, clinicians should thoroughly inform these patients about the potential long-term risks of increased wear and higher revision rates during preoperative counseling.

### Analysis of core symptoms and functional limitations influencing satisfaction

Complications following unicompartmental knee arthroplasty, such as implant loosening, wear, dislocation, and infection, are critical factors affecting long-term outcomes and implant survival, with aseptic loosening being a leading cause of revision [30]. However, this study suggests that early subjective patient dissatisfaction is more directly driven not by these traditional major complications, but by a constellation of clinical symptoms dominated by pain and functional impairments. Multivariate analysis identified walking pain (OR = 9.463), stair-related pain (OR = 8.783), knee instability (OR = 3.988), and restricted kneeling ability (OR = 0.128) as independent risk factors for dissatisfaction. The exceptionally high odds ratios for the various pain symptoms designate them as the strongest predictors of dissatisfaction.The etiology of these pain symptoms is diverse. Early postoperative pain may be related to surgical trauma, soft tissue inflammation, or the rehabilitation process. In contrast, persistent or new-onset pain in the mid- to long-term should raise suspicion for disease progression in the contralateral compartment or the patellofemoral joint. Our findings support the view that degeneration of the lateral compartment and the patellofemoral joint constitutes a significant source of postoperative pain [31]. UKA alters knee stress distribution. If pre-existing degeneration or malalignment is present in the lateral or patellofemoral compartments, postoperative load redistribution may accelerate pathology in these areas, causing walking or stair-climbing pain.Therefore, postoperative pain is not only a direct cause of dissatisfaction but may also serve as a clinical indicator of altered biomechanics or disease progression in other compartments of the knee.Beyond pain, the inability to achieve a full kneeling posture postoperatively emerged as a potent independent risk factor for dissatisfaction. This is far more than a simple functional metric; it carries significant biomechanical implications and reflects culturally specific activities of daily living. From a biomechanical perspective, achieving a comfortable kneeling posture requires deep knee flexion, often exceeding 130°-150°, and places high demands on patellofemoral and tibiofemoral joint coordination, quadriceps strength, and soft tissue balance [32]. Difficulty kneeling after UKA may indicate several issues: (1) prosthesis design or placement limiting maximum flexion, particularly due to posterior tibial component or polyethylene insert impingement; (2) abnormal patellofemoral tracking or progressive degeneration; or (3) postoperative capsular or soft tissue contracture leading to loss of deep flexion. Thus, ‘the ability to kneel’ serves as a high-order functional marker, reflecting surgical precision, prosthesis performance, and the state of the patellofemoral joint. Culturally, for populations with deep flexion demands in daily activities (e.g., kneeling for religious or domestic purposes, as seen in some Asian communities including the Hui ethnic group in our region), this functional limitation is particularly relevant for daily activities (e.g., domestic chores, sitting) and religious practices. This deeply ingrained functional need directly shapes patients’ postoperative expectations and their definition of surgical success, a notion supported by prior research linking high-flexion activities to satisfaction [33]Our study further identified a significant association between preoperative patellar morphology and both postoperative kneeling ability and satisfaction. Univariate analysis showed the highest dissatisfaction rate among patients with Wiberg type III patellae. This can be explained anatomically and biomechanically. The type III patella is characterized by a prominent lateral facet, a very small medial facet, and a medially displaced central ridge [34]. This morphology leads to abnormally concentrated contact stress on the lateral patellofemoral joint, predisposing to excessive lateral pressure syndrome (ELPS) and severe cartilage wear [35]. Concomitant changes in the Q-angle may further exacerbate patellar maltracking. Additionally, the type III patella is more prone to “tendofemoral contact” (contact between the patellar tendon and femur) during deep flexion, possibly an adaptive change to reduce focal cartilage pressure, but one that inherently limits high-flexion capacity. Consequently, patients with type III patellae are likely to encounter greater difficulty and discomfort during postoperative activities requiring deep flexion or high stress, such as stair climbing or kneeling. This persistent functional limitation, by impairing daily and culturally specific activities, indirectly lowers overall satisfaction. This underscores the importance of identifying Wiberg type III patellae during preoperative UKA assessment. Research suggests that correcting abnormal patellar morphology through techniques like patelloplasty may improve patellofemoral biomechanics and enhance postoperative functional outcomes and satisfaction [36]Finally, knee instability was also an independent factor for dissatisfaction. Although its OR was lower than that for pain symptoms, it warrants attention. The sensation of instability, which may stem from suboptimal ligament balance, improper implant positioning, or progressive joint laxity, can undermine patient confidence during activities, especially when turning or walking on uneven surfaces.

Based on this analysis, we propose that for patients with demands for deep flexion, preoperative assessment should include detailed inquiry into functional expectations and evaluation of patellar Wiberg classification. Intraoperatively, efforts should focus on achieving optimal implant matching, alignment restoration, and ligament balance, avoiding overstuffing or excessive tightness in the flexion gap. Postoperative rehabilitation should incorporate progressive deep flexion and stability training. Future prospective studies are needed to validate whether such a personalized surgical and rehabilitative approach, aimed at alleviating core symptoms and restoring specific functional needs, can systematically enhance patient satisfaction.Robotic navigation–assisted surgical technology has gradually matured, Recent studies have highlighted that improved alignment precision and reduced radiographic outliers are critical for enhancing patient-reported outcomes after UKA [37]; compared with conventional implantation, robotic-assisted UKA is associated with better Forgotten Joint Score-12 (FJS-12) and lower mid-term complication rates [38]. Furthermore, the type of surgical technology may affect long-term outcomes, such as the accuracy of posterior tibial slope [39]. Although our study did not directly address these technological factors, these findings support the notion that increased surgical precision and restoration of patient-specific alignment may contribute to improved patient satisfaction.

This study has several limitations. First, as a single-center retrospective study, it is susceptible to selection bias. Patients with incomplete data were excluded, and the comparisons of surgeon and prosthesis type are confounded by non-randomized assignment and differences in case mix. Second, satisfaction is a subjective measure that may be influenced by unmeasured patient expectations, psychological factors, and preoperative functional status (e.g., HSS, WOMAC, pain severity, range of motion), which were not collected in this cohort. Third, the primary symptoms (walking pain, stair-related pain, knee instability, kneeling difficulty) were assessed at the same final follow-up as satisfaction, precluding causal inference; these should be interpreted as concurrent correlates. Fourth, knee instability relied solely on patient-reported symptoms without standardized physical examination, and telephone follow-up may introduce recall bias. Fifth, the wide range of follow-up duration (20–102 months) and the use of only the last available assessment may obscure early dissatisfaction that resolved over time or late failures after the final visit. Sixth, inter-observer reliability for the Wiberg classification was not formally quantified, although the two evaluators reached consensus on discordant cases. Seventh, the comparison between mobile-bearing and fixed-bearing prostheses is subject to selection bias, as prosthesis choice may have been influenced by preoperative characteristics (e.g., deformity severity, ligamentous stability); propensity score matching was beyond the scope of this study. Eighth, the conclusions are drawn from a single center in Northwest China, and generalizability requires validation through multicenter studies. Finally, we did not perform stratified analyses by time period, implant type, or coronal alignment degrees; future studies with larger samples could explore non-linear relationships between alignment and satisfaction. Despite these limitations, the large sample size and detailed symptom assessment provide clinically relevant insights.

Therefore, the associations identified in the multivariable model cannot be interpreted as causal or predictive relationships; these factors should be understood as concurrent correlates of dissatisfaction rather than true independent predictors. Future prospective longitudinal studies are needed to determine whether addressing these symptoms preoperatively or early postoperatively would improve satisfaction. Future prospective research is also needed to confirm these risk factors and explore targeted intervention strategies.Patient dissatisfaction after UKA is independently associated with walking pain, stair-related pain, knee instability, and inability to kneel fully. Kneeling difficulty is particularly significant within specific cultural contexts. Preoperative counseling should address these modifiable factors.

## Data Availability

The datasets generated and/or analysed during the current study are not publicly available due to participant confidentiality of this research but are available from the corresponding author on reasonable request.

## Abbreviations

UKA: Unicompartmental Knee Arthroplasty
KOA: Knee Osteoarthritis
TKA: Total Knee Arthroplasty
BMI: Body Mass Index
HKA: Hip-Knee-Ankle Angle
PTS: Posterior Tibial Slope
HSS: Hospital for Special Surgery Knee Score
WOMAC: Western Ontario and McMaster Universities Osteoarthritis Index
FJS-12: Forgotten Joint Score-12
ASA: American Society of Anesthesiologists
OR: Odds Ratio
CI: Confidence Interval
ELPS: Excessive Lateral Pressure Syndrome
